# Disrupting Viral Persistence: CRISPR/Cas9‐Based Strategies for Hepatitis B and C Treatment, and Challenges

**DOI:** 10.1111/jcmm.70986

**Published:** 2026-01-08

**Authors:** Meng‐Fan Li, Akmal Zubair, Safa Wdidi, Shan He

**Affiliations:** ^1^ Food Science School Guangdong Pharmaceutical University Zhongshan China; ^2^ Department of Biotechnology Quaid‐i‐Azam University Islamabad Pakistan; ^3^ Faculty of Medical Sciences Laboratory, Oncology Research Center University of Shendi Shendi Sudan; ^4^ Faculty of Science, Technology and Engineering Charles Darwin University Casuarina Northwest Territories Australia

**Keywords:** cccDNA, CRISPR/Cas9, gene editing, HBV, HCV

## Abstract

Hepatitis B and C viruses (HBV and HCV) remain among the leading causes of liver disease worldwide. Current antiviral drugs, such as nucleotide analogues (NAs), can reduce the replication of new HBV and HCV infections but cannot completely eliminate chronic infections. This is primarily because a stable form of viral DNA, known as covalently closed circular DNA (cccDNA), persists in liver cells and continues to sustain the infection. In recent years, the CRISPR/Cas9 gene‐editing system has emerged as a powerful tool for precisely cutting and inactivating specific DNA sequences. Due to its efficiency and ease of use, researchers have applied CRISPR/Cas9 in numerous studies to directly target and disrupt the HBV genome, demonstrating promising antiviral effects in both cell cultures and animal models. Targeting multiple sites within the HBV genome has been shown to further enhance its effectiveness, paving the way for potential combination therapies aimed at disabling both cccDNA and HBV and HCV DNA integrated into the host genome. Despite its potential, CRISPR/Cas9 still faces significant challenges before clinical application, most notably the risk of off‐target effects—unintended cleavage of non‐target DNA sequences—and the difficulty of delivering the system efficiently into liver cells in vivo. Future progress will depend on improving the tool's precision, efficiency, flexibility and delivery methods. In this review, we explore recent advances in designing guide RNAs (gRNAs) for targeting HBV and HCV, as well as the delivery systems used to transport CRISPR/Cas9 into cells. We also discuss the remaining challenges and potential strategies for advancing CRISPR/Cas9 from the laboratory toward a viable clinical cure for HBV and HCV.

## 1 Introduction

Approximately 500 million people worldwide are affected by chronic viral hepatitis, which can lead to cirrhosis, cancer and liver failure [1]. Ninety percent of primary liver cancers are hepatocellular carcinomas (HCCs), making liver cancer the third most lethal type of cancer overall. Solid tumours, including HCC, are notoriously difficult to detect and treat due to their complexity and the variability of cancer genetic profiles [2]. Hepatocellular carcinoma (HCC) is more common in individuals with a history of chronic hepatitis B, delta or C infections [3]. The importance of persistent viral infection in the aetiology of HCC underscores the need to understand the immunological and viral mechanisms driving HCC pathogenesis. Hepatocarcinogenesis may be initiated by hepatitis B virus (HBV), hepatitis C virus (HCV) and hepatitis D virus (HDV) through shared processes, including oxidative stress induced by immunological and viral proteins, hepatic inflammation accompanied by impaired antiviral immunity and the deregulation of cellular signalling pathways by viral proteins [4]. HBV infection is characterised by DNA integration, leading to genomic instability and metabolic reprogramming, which results in steatosis. HBV infection is a major global public health concern, affecting approximately 2 billion people worldwide with evidence of past infection [5]. Approximately 350 million individuals worldwide are chronically infected with this virus, making it one of the most widespread diseases in humans [6]. The probability of contracting chronic hepatitis B (CHB) infection after exposure appears to increase with age [7]. Infection during the first year of life increases the likelihood of developing (CHB) by 90%, whereas infection later in infancy raises the risk by 20% to 30%. Exposure in adulthood reduces the risk to less than 1% [8]. Cirrhosis serves as a precursor to HCC, which can develop from persistent infections. The leading cause of HCC worldwide, affecting nearly 50% of all cases, is chronic hepatitis infection [9]. A notable finding demonstrating an etiological link between (CHB) and (HCC) is the increased incidence of HBV surface antigen (HBsAg) positivity in HCC patients [10]. Individuals with CHB have a 20‐fold increased risk of developing HCC, even in the absence of cirrhosis. Research conducted by the REVEAL‐HBV study demonstrates a definitive link between circulating HBV DNA levels and the risk of (HCC) [11]. HBV‐DNA integration into hepatic cells increases the relative risk of (HCC) in HBsAg carriers by one hundredfold compared to non‐carriers [12].

The hepatitis C virus (HCV) causes hepatitis C, a viral infection that primarily affects the liver. Some individuals may remain asymptomatic during the initial infection; however, later symptoms such as jaundice, dark urine and abdominal discomfort are common [13]. The virus progresses to cirrhosis over many years, remaining dormant in the liver and causing a latent illness with few outward symptoms during that time. Hepatitis C, a liver disease, remains a major health problem despite the approval of several direct‐acting antiviral regimens, causing over 400,000 deaths each year [14]. The proteases produced by the HCV can generate 15 unique polypeptides from a single precursor protein [15]. The structural heterogeneity of the HCV arises from its multiple genomes and other components that form distinct regions. The HCV genome encodes several proteins, including two glycosylated peptides (E1 and E2) and seven non‐capsid (NC) peptides, totalling approximately 4000 amino acids. A heterodimer of E1 and E2 is formed on the viral envelope. The receptor‐binding domain (RBD) on E2 influences the interaction with entry receptors. The primary function of E2 is the neutralisation of antigens [16]. Proteins lacking structural domains include ferroportin, NS2, NS3, NS4A, NS4B, NS5A, NS5B and others [4]. The components required for HCV RNA translation and the synthesis of the NS3‐NS5B polyprotein are found in various NS polymerase complexes [17]. NS5B, an RNA‐dependent RNA polymerase that directly regulates RNA synthesis, is one of these proteins [18]. Chronic HCV and HBV infections remain the leading causes of liver disease worldwide. Modern HCV therapy is dominated by DAAs that specifically inhibit viral enzymes. DAAs include protease inhibitors (targeting NS3/4A; e.g., boceprevir, simeprevir), NS5A inhibitors (such as ledipasvir and daclatasvir) and polymerase inhibitors (including sofosbuvir and dasabuvir). By combining two or more DAAs for 8 to 24 weeks, treatment achieves a sustained virologic response (SVR, equivalent to a cure) in over 90%–95% of patients. In fact, the World Health Organization (WHO) now recommends that all adults and children aged 3 years and older receive pan‐genotypic DAA regimens (such as sofosbuvir+velpatasvir or sofosbuvir+daclatasvir), which have minimal toxicity and can cure most cases in one 12‐ to 12‐revolution in HCV care has made hepatitis C a curable disease in principle. However, access remains a major barrier: only about 36% of people with HCV know their status, and roughly 20% of infected individuals worldwide had received DAA treatment by 2022. Additional challenges include potential drug–drug interactions (important in patients on particularly important for and viral resistance in the small fraction of non‐responders). High drug prices have also limited uptake, though generic DAA combinations, such as sofosbuvir+daclatasvir, have dramatically cut costs in many low‐ and middle‐income countries. In summary, DAAs can achieve > 95% cure rates, but global elimination hinges on rates exceeding 95%, expanding testing, improving access and maintaining effective treatment delivery.

Chronic (HBV) therapy relies on two primary strategies: nucleoside and nucleotide analogs (NAs) and interferon‐alpha [19]. First‐line NAs, primarily tenofovir (administered as tenofovir disoproxil or tenofovir alafenamide) and entecavir, are potent reverse transcriptase inhibitors [20]. These drugs rapidly suppress HBV DNA, reduce liver inflammation and fibrosis and improve long‐term outcomes. For example, the World Health Organization (WHO) notes that tenofovir or entecavir therapy “can slow the progression of cirrhosis, reduce cases of liver cancer and improve long‐term survival” in HBV patients. In practice, NA therapy is usually lifelong because NAs do not eliminate the viral covalently closed circular DNA (cccDNA) reservoir in the hepatocyte nucleus [21]. Consequently, cessation of NAs almost invariably leads to viral rebound. Interferon‐alpha (particularly pegylated IFN‐α) can be administered for a fixed course and stimulates immune‐mediated clearance. A minority of patients (approximately 3%–12%) achieve sustained hepatitis B surface antigen (HBsAg) loss after peg‐IFN therapy, but most do not. Furthermore, interferon therapy has significant side effects, including flu‐like symptoms, cytopenias and neuropsychiatric effects, that limit its use [22]. In practice, only a subset of HBV patients qualifies for treatment. Global estimates reveal significant care gaps: as of 2022, only approximately 13% of HBV carriers were diagnosed, and just about 3% were receiving treatment [23]. However, most patients require indefinite NA therapy, which typically results in only functional control, as an HBsAg‐negative cure is very rare. Because current therapies suppress but do not universally cure HBV, and because there is no vaccine yet available to prevent HCV, researchers are investigating genome‐editing approaches as potential cures [24]. CRISPR‐Cas systems can be programmed to target viral nucleic acids within infected cells. For HCV, an RNA virus, RNA‐targeting nucleases such as CRISPR‐Cas13 have shown promise. In one study, researchers designed Cas13a with guide RNAs targeting the conserved internal ribosomal entry site (IRES) of HCV; delivery of Cas13a into liver cells resulted in significant inhibition of HCV replication and viral protein expression, with minimal toxicity. This proof‐of‐principle suggests that CRISPR‐Cas13 “molecular scissors” could be used to degrade HCV RNA in vivo, although delivery methods and off‐target effects must be optimised before clinical application [25].

For (HBV), which maintains its genome as nuclear covalently closed circular DNA (cccDNA) and often as integrated DNA, DNA‐targeting CRISPR systems are under investigation [26]. The classical CRISPR‐Cas9 nuclease can be directed to conserved HBV sequences to induce double‐strand breaks in cccDNA, leading to its destruction or inactivation [27]. More recently, base editors, CRISPR‐derived enzymes that make precise single‐base changes without causing double‐strand breaks, have been applied. In a recent mouse model, researchers used a cytosine base editor with two guide RNAs to introduce stop‐codon mutations in the HBV surface (S) and precore genes [28]. This multiplexed base editing resulted in substantial reductions in viral markers: over a 2‐log (less than 1% of baseline) decrease in serum HBsAg, with 6 of 9 mice showing complete loss of detectable HBsAg, and over a 3‐log drop in HBV DNA, with no viral rebound after treatment [29]. These edited animals effectively lost HBV antigen production, demonstrating that permanently disabling the viral genome is possible. Overall, emerging studies indicate that CRISPR/Cas targeting of HBV in cells or animal models can irreversibly silence cccDNA or integrated HBV genes [30]. Although still preclinical, such approaches could 1 day yield a “functional cure” characterised by sustained HBsAg loss for chronic HBV infection as shown in Table 1.

**TABLE 1:** Lists popular online tools for designing CRISPR guide RNAs (gRNAs), along with their websites, purposes and key features.

Tool name	Website	Purpose	Features
CHOPCHOP	https://chopchop.cbu.uib.no/	A convenient gRNA designer with support for many of the existing CRISPR systems	Evaluates secondary configuration in the gRNA on‐target efficiency and off‐target projections
CRISPOR	http://crispor.gi.ucsc.edu/	A broadly utilised device for the design of detailed gRNA and non‐specific projection	Estimates off‐target regions, offers specificity scores and structural depiction and constructs gRNA models
E‐CRISP	http://www.e‐crisp.org	Interface for creating gRNAs with accurate scoring for numerous organisms	Delivers on‐target effectiveness and off‐target potential examination
CRISPR‐ERA	http://crispr‐era.stanford.edu/	Formulate gRNAs with extensive examinations for different CRISPR systems	Measures off‐target effects, recognises specific regions and values gRNA efficacy and precision
Cas‐Designer	http://www.rgenome.net/cas‐designer/	Stable fsramework that supports several CRISPR systems	Extensively assesses both off‐target potential and on‐target actions
CRISPRdirect	https://crispr.dbcls.jp/	Easy tool for formulating gRNAs with little non‐specific impacts	Utilising an easy interface to detect target regions rapidly
CCTop	http://crispr.cos.uniheidelberg.de	Formulate CRISPR/Cas9 gRNAs with greater precision and fewer non‐specific outcomes	Projects off‐target outcomes, detects target sites and scores gRNA efficiency with a convenient interface
GuideScan	https://guidescan.com/	Focuses on minimising non‐specific impacts and enhancing on‐target activities	Detects and rates putative gRNAs using accurate off‐target prediction

*Note:* Each tool offers unique strengths, such as off‐target prediction, efficiency scoring and support for different CRISPR systems, helping researchers select precise and effective gRNA sequences for their experiments.

## 2 General Mechanisms and Development of CRISPR Systems

CRISPR/Cas technology has evolved from enhancing bacterial immunity to becoming a powerful gene‐editing tool, significantly streamlining genomic research in mammals and enabling more precise gene modifications. A CRISPR/Cas system typically consists of an RNA‐guided nuclease (RGN) and a guide RNA (gRNA) [31, 32]. The CRISPR gene family in bacteria encodes numerous short repeats and spacers. The immune system uses these spacers as a “blacklist,” derived from foreign DNA sequences acquired by the bacteria [33]. The conversion of short direct repeats containing palindromic sequences into a hairpin structure facilitates the formation of functional CRISPR RNA (crRNA) and trans‐activating CRISPR RNA (tracrRNA) [34, 35]. Various activities depend on operon expression systems, including target cleavage, spacer acquisition, crRNA processing and others. The highly conserved CRISPR‐associated (Cas) loci are located near the CRISPR genes. The Cas RNA‐guided nuclease (RGN) uses crRNA and tracrRNA to recognise and eliminate foreign sequences, thereby providing protection against infections [36]. The CRISPR/Cas system can modify specific genomic regions in mammalian cells due to its RNA‐guided targeting and cleavage capabilities [37, 38]. Evidence from multiple cell lines and animal models suggests that customised CRISPR/Cas systems effectively regulate gene expression, identify functional gene signatures and correct disease‐associated mutations [39, 40].

## 3 Application of CRISPR/Cas Technology to Treating HCV Infection

The bacterium *Streptococcus pyogenes* (Sp) has been extensively studied for its ability to target DNA using its CRISPR/Cas9 system. However, insufficient research has been conducted on the potential medical applications of CRISPR/Cas systems for RNA targeting. The infectious bacterium *Francisella novicida* (Fn) possesses a CRISPR/Cas9 system that, as noted by [41] may endonucleolytically cleave an endogenous bacterial transcript. The canonical pathway unexpectedly identifies one of the additional genomic loci that the *F. novicida* CRISPR/Cas9 system can target. Short CRISPR/Cas‐associated RNA (scaRNA), a unique guide RNA, mediates the RNA‐inactivating activity. Similar to crRNAs, scaRNA interacts with the trans‐activating crRNA (tracrRNA) to form the targeting complex with FnCas9. By engineering a guide RNA (rgRNA) that targets the HCV genomic sequence, FnCas9 was reprogrammed to function similarly to SpCas9 [42]. By attaching a tracrRNA motif to a single‐stranded scaRNA, the rgRNA exhibited structural similarity to sgRNA. FnCas9 can be directed to preferentially bind viral RNA by generating scaRNAs derived from the HCV genome. The researchers successfully suppressed genes associated with HCV replication using cell culture models. Although endonuclease domains often play a role in regulating gene expression within the FnCas9 system, they were found to be unnecessary for HCV rgRNA‐induced suppression. Subsequent studies demonstrated that rgRNA‐containing FnCas9 inhibited translation, thereby reducing HCV gene expression; however, this research was conducted in a controlled laboratory environment [43]. Additionally, the researchers demonstrated that FnCas9 technology may be capable of preventing viral replication by targeting the negative‐sense RNA strand of HCV. The ability of FnCas9 to bind nucleic acids was essential for gene suppression, even though its nuclease domains were not required. Although the exact mechanism by which FnCas9 induces gene silencing remains unknown, this additional RNA‐inactivating capability expands the potential applications of CRISPR/Cas9 technology. Table 2 shows the preclinical trials of CRISPR/Cas9 for Hepatitis B and C [45].

**TABLE 2:** Summarises common in vitro and in vivo delivery methods for CRISPR/Cas9 in HBV gene editing, outlining their principles, key features and research applications.

Category	Methods	Features	Applications
In vitro delivery	Chemical Transfection	Chemical compounds promote the absorption of CRISPR/Cas9 complexes by cells	HBV gene editing in cell lines is easy and affordable; however, it is not as effective as other techniques
Electroporation	For the entry of CRISPR/Cas9, short electric pulses create transient pores in the cell membrane	High transfection potential for difficult‐to‐transfect cells, appropriate for altering the HBV gene in primary hepatic cells and other tough cell types	
Microinjection	A fine needle is utilised for the direct transfusion of CRISPR/Cas9 elements into separate cells	Although labor‐intensive and technically complex, this precise approach is appropriate for HBV gene editing in primary cells and single‐cell manipulation experiments	
Virus‐mediated delivery (AAV, Lentivirus)	Viral plasmids are used to transfer CRISPR/Cas9 genes into cells or target tissues for steady integration	Generation of it is ideal for producing stable HBV knockout cell lines and is appropriate for long‐lasting HBV gene editing research	
Nanoparticles	Nanoparticles enclose CRISPR/Cas9 mRNAs, aiding their delivery and adsorption into cells.	Effective delivery of CRISPR/Cas9 for HBV gene editing in a variety of cell types, appropriate for high‐throughput testing and functional research.	
In vivo delivery	Virus‐mediated transfusion (AAV, Lentivirus)	Viral plasmids are effective carriers for transporting CRISPR/Cas9 elements to target tissues	Providing long‐lasting expression and stable integration, CRISPR/Cas9 in vivo delivery for HBV gene engineering in animal models
Nanoparticles	CRISPR/Cas9 components are encapsulated for efficient transfusion to target tissues	The hepatic tissues of animal models are used for clinical translation and, due to biocompatibility, for in vivo delivery of CRISPR/Cas9 for HBV gene modification	
Hydrodynamic Injection	Fast infusion of a significant volume of DNA solutions causes temporary DNA transfer in the liver.	CRISPR/Cas9 delivery for editing in the HBV gene in animal models is appropriate for transient expression and quick evaluation of gene editing effectiveness	
in vivo electroporation	Short electric pulses promote the absorption of CRISPR/Cas9 complexes by tissues	In vivo delivery of CRISPR/Cas9 for editing of the HBV gene in hepatic tissues of animal models provides effective transfection without viral vectors	
Microinjection	Direct infusion of CRISPR/Cas9 elements into targeted embryos or tissues	Accurate transport of CRISPR/Cas9 for HBV gene editing in animal models that works well for research that needs single‐cell modification or spatial control	

*Note:* It covers chemical, physical and viral approaches, highlighting their suitability for different cell types, precision levels and experimental goals [44].

## 4 Utilisation of CRISPR/Cas to Directly Target Viral Genomes to Inhibit HBV

**FIGURE 1:**
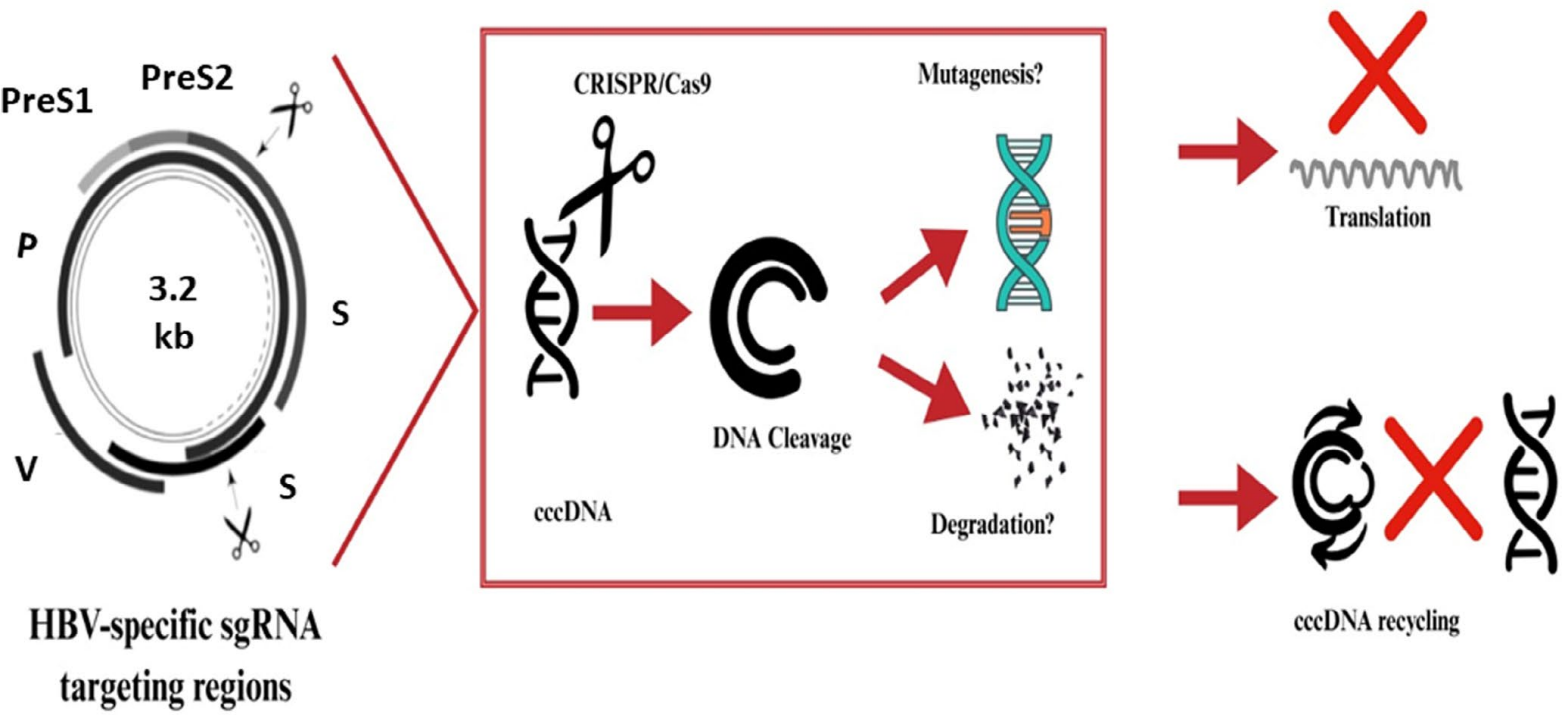
Schematic representation of CRISPR/Cas9‐mediated targeting of (HBV) covalently closed circular DNA (cccDNA). Specific single‐guide RNAs (sgRNAs) are designed to recognise HBV genomic regions such as preS1, preS2 and X. Cas9‐induced (DSBs) lead to cccDNA mutagenesis or degradation, thereby inhibiting viral gene translation and preventing cccDNA recycling essential for persistent infection.

Owing to its straightforward framework, remarkable adaptability and ease of use, CRISPR/Cas9 has attracted the interest of many researchers as a promising method for targeting pathogenic viral genomes [46, 47]. In 1987, CRISPR regions, recurrent segments of DNA, were discovered in *Escherichia coli* , though their roles and origins were initially unclear. In 2012, it was demonstrated that the CRISPR/Cas complex, along with the effector protein Cas, provides adaptive immunity in bacteria. This immunity manifests as resistance to plasmids and phages, which are two forms of foreign genetic material [48]. The CRISPR/Cas system is used to cut the HBV genome as shown in Figure 1.

Each of the six distinct CRISPR/Cas technologies is named after a component of the effector complex. The Type II CRISPR/Cas system consists of three elements: CRISPR RNA (crRNA), the nuclease Cas9 and trans‐activating CRISPR RNA (tracrRNA), which activates the CRISPR mechanism [49, 50]. A linker loop sequence can be used to combine crRNA and tracrRNA into a single guide RNA (sgRNA) while preserving their functional efficacy. The crRNA confers target specificity to the CRISPR/Cas complex through nucleotide complementarity upstream of the protospacer adjacent motif (PAM). This compact region, approximately three to eight base pairs in length, is essential for Cas9's initial recognition of the target DNA. Upon recognition, the double‐stranded DNA (dsDNA) unwinds, forming an R‐loop characterised by an RNA–DNA hybrid and exposed single‐stranded DNA (ssDNA). The two endonucleolytic domains of Cas9, RuvC and HNH, introduce precise DSBs approximately three to four base pairs upstream of the PAM. The resulting DSBs are repaired through a restoration procedure known as joining (NHEJ). The editing (NHEJ) pathway often generates insertions and deletions (indels). Genome editing effects can occur at the intended site (“on‐target”) or in non‐targeted off‐target sites with nucleotides similar in sequence, though with significantly lower efficiency. For these reasons, the applications of the system have been modified and engineered to enhance the intended effects and reduce non‐specific effects. To minimise off‐target DSBs, the nuclease domain can be inactivated, producing Cas9 variants. DNA in ssDNA within the R‐loop can be targeted by tools, and editing tools for precise nucleotide modifications can be regulated through CRISPRi and CRISPRa: CRISPR interference (CRISPRi) and CRISPR activation (CRISPRa).

## 5 Double‐Strand Break DSB‐Based CRISPR/Cas

CRISPR technology enables the modification of multiple loci within the HBV genome. The compact genome size, along with the prevalence of highly conserved sites, facilitates the design of effective guide RNAs (gRNAs) capable of targeting the genome. Furthermore, due to the substantial genomic overlap, insertions and deletions that repair DSBs may collectively inhibit HBV replication. The Pol protein and the HBx open reading frame (ORF) can be affected at different stages of the viral life cycle by factors such as HBs ORF cleavage and the targeting of Enhancer II (EnhII), respectively. The feasibility of cleaving HBV genomes using CRISPR/Cas9 has been investigated in numerous studies employing various HBV replication models. Many researchers, particularly Martinez et al. [47, 51] have consolidated this data. Genomic RNAs (gRNAs) targeting highly conserved regions of the HBV genome were developed by screening stable, easily transfectable cell lines. The efficacy of the primary gRNAs was further validated in more advanced and representative culture systems, including primary human hepatocytes (PHHs), HBV‐infected HepG2‐NTCP cells and in vivo in transfected or immunocompromised humanised transgenic liver mice. Additionally, the study included both targeted and comprehensive off‐target assessments [51].

This review examines recent studies that (i) employ CRISPR‐Cas9 double‐strand break technology to modify HBV genes, thereby enhancing our understanding of the virus's biology, and (ii) utilise clinically relevant methods to deliver transiently expressed CRISPR/Cas9 components, such as ribonucleoprotein (RNP) complexes, mRNA‐expressing gene editors or both. Due to its role in cccDNA transcription and its association with HBV‐induced HCC, the HBx open reading frame (ORF) is a critical target for CRISPR/Cas9 [52] In a study using the HepG2.2.15 cell line and HepG2 cells transfected with a plasmid expressing HBV, researchers applied CRISPR/Cas9 to precisely target the HBx loci. The findings demonstrated a reduction in cccDNA levels, decreased secretion of HBsAg and lowered HBx mRNA and protein expression [52]. Inhibiting cell motility, chemotactic migration and invasion by targeting the HBx open reading frame resulted in a reduced tumorigenic potential of HBx in HepG2.2.15 and Hep3B cells genetically engineered with an HBV vector. Numerous genes associated with cancer stemness and the epithelial–mesenchymal transition (EMT) were downregulated following HBx knockdown, as demonstrated by mechanistic studies [52].

The genes encode E‐cadherin (CDH1), fibronectin, CD133, alpha‐smooth muscle actin (α‐SMA), vimentin, N‐cadherin (CDH2) and Thy1. A comprehensive analysis of genetic variations affecting metabolism, histone modifications, viral infections and signal transduction pathways was conducted using transcriptomics and RNA sequencing data. Reductions in spheroid number, size, migration, HBV mRNA expression and cccDNA levels were observed in both two‐ and three‐dimensional spheroid models, supporting the concept of HBx targeting [53]. The research elucidates the potential impacts of HBx on the host transcriptome and the mechanisms through which CRISPR‐Cas9 editing may correct these modifications. Gene regulation is a complex biological process. This is the first study using in vitro models to examine the effects of double‐strand break (DSB) cleavage in the HBx region; however, it is not the only study to conduct this analysis. This research has provided further insights into HBV characteristics [54, 55].

The CRISPR/Cas9 components can be delivered into host cells using either viral or non‐viral methods [56]. The half‐life of gene‐editing reagents delivered using RNP‐based methods is shorter than that of plasmid or viral vectors, due to the rapid degradation of RNPs by endogenous nucleases and proteases. Delivering CRISPR/Cas9 components via RNPs is advantageous because their brief lifespan reduces off‐target mutagenesis [57]. Targeting the cccDNA‐like plasmid (rcccDNA) with ribonucleoproteins (RNPs) has been achieved using *Streptococcus thermophilus* Cas9 (StCas9) and HBV‐specific guide RNAs (gRNAs) [57]. Utilising StCas9 to recognise an extended (PAM) sequence offers several advantages [58].

The authors demonstrated that methylation may reduce the efficacy of gRNA in a cleavage experiment using highly methylated rcccDNA (meth‐rcccDNA) in vitro. Increasing the amount of StCas9 restored cleavage efficiency, highlighting the importance of gene‐editor dosage in mitigating the negative effects of methylation. They also investigated the effect of transfecting HepG2‐NTCP cells with both methylation‐specific and unmethylated rcccDNA. While in vitro studies suggest that DNA methylation influences the cleavage efficiency of StCas9 RNPs, similar decreases were observed in both chemically methylated and unmethylated rcccDNA, indicating otherwise.

The authors conducted further research on the role of rcDNA in HBV recurrence. This study involved infecting nucleofected HepG2‐NTCP cells with RNP 7 days after CMV contamination. The impact on virological factors was assessed at two post‐infection time points: 14 days (dpi) and 24 days (dpi). Levels of cccDNA and HBsAg increased over time, indicating that HBV could reemerge in samples treated with RNP therapy. No reduction in cccDNA was observed at 7 days post‐infection when StCas9 was delivered alone. The effects of target mutations on the selected trait may be clarified by the results of specific breakage cleavage using RNP (such as indels) in this hypothetical cccDNA. Preemptive reduction of the rcDNA reservoir before targeting cccDNA with Cas9 RNP may enhance treatment of HBV infection [57, 59]. The rebound at 17 days post‐infection (dpi) was less effective, resulting in a reduction of cccDNA levels when combined with LAM. Previous studies suggested that cccDNA maintenance might persist indefinitely without continuous replenishment; however, recent findings challenge this assertion [59].

The findings indicate that viral rebound after CRISPR targeting and the de novo synthesis of rcDNA to cccDNA replenishment is primarily associated with the HepG2‐NTCP framework. A broader cohort of patients receiving nucleos(t)ide analogues (NAs) may benefit from CRISPR/Cas9 therapy, depending on future research into the effects of the RNP + NA combination in primary hepatocytes, in vivo and regarding off‐target effects. Subsequent work has focused on determining whether multiplexing gRNAs increases the susceptibility of cccDNA to degradation or leads to the permanent disruption of multiple open reading frames (ORFs) [54]. Fei et al. [54] endorse the use of lentivirus‐mediated dual gRNAs targeting HBV DNA. We evaluated 10 gRNAs targeting HBV in HepG2 cells transfected with HepAD38 or rcccDNA to determine the optimal conservation rate across genotypes A and H. The researchers found that these gRNAs significantly reduced cccDNA levels, total HBV DNA, intracellular 3.5 kb RNA and extracellular HBsAg and HBeAg while maintaining cell viability. A 2‐by‐2 multiplexing approach using gRNAs targeting the S/Pol, Pol/X initiator sequence or precore regions was employed to reduce viral parameters in HepG2‐NTCP cells infected with HepAD38 and HBV.

Zhang et al. [60] targeted overlapping genotype‐independent HBV DNA fragments in the C/Pol, Pol/preS1, S/Pol and Pol/HBx regions using artificial gRNA/Cas9 RNP technology. Following screening in HepG2 cells, two gRNAs directed at Pol/S and Pol/HBx were selected for further validation (see Section 2.15). The HepDE19 and HepG2‐NTCP cell lines showed a significant decrease in cccDNA levels after stable transfection with gRNA or HBV. According to the results of MTT assays, Annexin V staining and 7‐AAD accumulation tests, gRNA/Cas9 RNPs exhibited no adverse effects on human hepatic cell types [60]. The studies showed no effect on plasma membrane integrity, cell death or cell survival. Our results indicate that synthetic gRNA/Cas9 RNPs may inhibit HBV replication; however, further in vivo studies are necessary to evaluate its comprehensive efficacy against HBV and the immune system's response [61]. Gene transfer is just one of several potential therapeutic applications of extracellular vehicles (EVs), which are nanoscale biological entities. The suppression of endosomal egress may impede the passage of cargo to target cells. Zeng et al. [29] successfully addressed this problem by encapsulating extracellular vesicles with VSV‐G and incorporating Cas9/gRNA ribonucleoprotein. The modified EVs significantly altered the HBV DNA.

To enhance the payload delivery of electric vehicles (EVs), a Cas9 enzyme was conjugated with a Cryptochrome 2 (CRY2) peptide and a CRY2‐ligand protein, CIBN, which carried a membrane‐bound acetylation label. The light‐activated CRY2‐CIBN heterodimerization rapidly targets Cas9 to membranous structures, thereby improving the encapsulation of gRNA/Cas9 ribonucleoproteins (RNPs) into extracellular vesicles. The presence of a fusion‐inducing VSV‐G peptide on extracellular vesicles reduced endosomal entrapment in recipient cells, facilitating the intranuclear transport of RNPs within these cells. After a 48‐h incubation of HepG2‐NTCP cells with EVs, concentrations of HBV DNA, HBsAg and extracellular HBeAg were significantly decreased. Additionally, reductions in intracellular HBcAg and covalently closed circular DNA (cccDNA) levels were observed. The HBV genome was enzymatically cleaved in vitro using dual gRNAs. In mice infected with replicating HBV transfected with a 1.2× HBV replication unit of genotype C, a notable decrease in HBsAg and HBeAg levels was observed, along with reduced intrahepatic HBcAg staining 7 days post‐infection [29]. Further off‐target investigations are necessary to ensure the safety of this innovative delivery technique before its use in clinical trials; nonetheless, it shows potential by reducing immunogenicity and mitigating some problems associated with Cas9 administration. It is essential to acknowledge the possible limitations of dual‐gRNA targeting of cccDNA. In vivo HBV genotype C replicon [62] and dual‐gRNA‐stimulated breakage of the HBV vector [63] provide methods that effectively eradicate the HBV genome (DNA sequences ranging from 665 to 702 base pairs). It is crucial to assess the potential of these fragments to produce minor, transcriptionally active CRISPR episomal variants when considering a therapeutic approach for HBV.

Yu and associates [64] conducted a proof‐of‐concept experiment focusing on conserved regions of HBV variants A–D, using a method and delivery vehicle suitable for therapeutic administration. They identified the optimal gRNA combination for reducing HBV proteins (HBsAg and HBeAg) by employing AAV‐HBV1.04‐transduced AML12 murine cultures and HuH7 cells transplanted with an experimentally synthesised cccDNA‐like polymer. The G17+G60 combination was determined to be ideal for human studies, which involved administering Cas9‐encoding mRNA via SM‐102 lipid nanoparticles (LNPs) to transiently produce the Cas9 protein [64]. gRNA g17 has a double‐stranded DNA (dsDNA) cleavage site similar to one documented in previous research, highlighting the importance of these conserved regions in regulating HBV replication [65]. The verification of effective biological distribution and mRNA synthesis encoding GFP in the hepatic tissue of the C57BL/6 murine model at 6 h post‐exposure confirmed the efficacy of the SM‐102 LNP delivery technique. Following administration of Cas9 mRNA and HBV‐targeting gRNAs (g17+g60), infected tree shrews exhibited a significant reduction in viral markers, including intrahepatic HBc and HBsAg, HBV DNA, released HBsAg, HBeAg and cccDNA levels. This decrease did not affect the expression of intrahepatic immunostimulatory genes (IFNα, IRF3 or ISG15) or ALT/AST levels. Genetically modified mice harbouring a 1.28‐mer Gen A HBV genome showed reduced HBV RNA levels. A novel approach has been developed to selectively label cccDNA and combined HBV DNA while minimising the risk of (HCC): amplified products from specific putative host genes subjected to next‐generation sequencing (NGS). This method was evaluated in transgenic mice and tree shrews, demonstrating minimal off‐target effects. Notably, after HBx targeting, the HBV restriction factor SMC5/6 may reemerge in the liver. This is considered a favourable development, as it may help inhibit unmodified cccDNA molecules [64]. The traditional Cas9 nuclease‐mediated editing method can still produce unexpected results, despite the increasing availability of enhanced Cas9 proteins and guide RNAs (gRNAs) designed to minimise off‐target effects and optimise specificity. Off‐target editing can cause significant genomic alterations, such as large deletions or truncations, which may be harmful and challenging to detect [62]. Chromosomal breaks and rearrangements, known as chromothripsis, can lead to complex genomic alterations and, consequently, genotoxicity [63]. When standard Cas9 cleaves integrated HBV DNA, unintended consequences may occur. Employing gene editing techniques that avoid the formation of (DSBs) could mitigate these issues. The application of CRISPR therapy for hepatitis is summarised in Table 3.

**TABLE 3:** CRISPR‐based therapeutic studies in viral hepatitis explore the use of gene‐editing technologies, particularly CRISPR/Cas systems, to target and disrupt viral genomes or host factors essential for viral replication.

Hepatitis viruses	Therapeutic targets	In vitro or in vivo models	Methods and vectors	Gene‐editing efficiency (%)	Therapeutic effects	References
HBV1.2	XCp (1742–1764), P1 (1292–1314)	Huh7 cells, mouse models with a hydrodynamic shot of 1.2× HBV vectors	The sgRNA and human codon‐optimised Cas9 (hCas9) plasmids are administered via hydrodynamic injection (HDI) in vivo and lipofectamine in vitro, respectively	In vitro: 25.6% In vivo: about 5%	Decline in rcDNA and cccDNA	[66]
HBV concentrated 100‐fold from the culture broth of HepAD38 cells	ENII/CP/X (2987–3006; 3048–3067; 3062–3081), Pre‐C (2–21)	HepG2 cells translating sodium taurocholate co‐transporting polypeptide (NTCP)	Lentivirus‐carried sgRNA and CW‐Cas9 vectors (in vitro)	In vitro: over 60%	Eightfold HBV suppression	[65]
HCV	RNA of HCV	Huh‐7.5 cells	Lipofectamine‐carried FnCas9/rgRNA vector (in vitro)	Unshown	Suppression of HCV peptide synthesis	[42]
HBV1.3	X (1523–1542; 1681–1700)	Huh7 cells, HepG2.2.15 cells, mouse models with a hydrodynamic infusion of precccDNA vector	PX330 carried by lipofectamine (in vitro) and HDI (in vivo)	In vitro: 44.2% (gRNA1) and 34.2% (gRNA2) In vivo: unshown	Inhibition of intracellular cccDNA (with > 60% reduction) and viral multiplication	[67]
HBV 1.3	S1 (357–376), X1 (1406–1425)	HepG2 cells, HepG2.2.15 cells, mice with HBV‐transgene (Tg)	hCas9 vector and sgRNA vector carried by PEI (in vitro) and HDI (in vivo)	In vitro: displayed In vivo: over 50%	A reduction of more than 50% in HBsAg and 58%–75% in HBV DNA modifications	[68]
HBV 1.3	ORF S, core, polymerase, X	HepG2 cells, HepG2.2.15 cells and immunodeficient mice (NRG) with 1.3× HBV plasmids injected hydrodynamically	hCas9/sg HDI (in vivo) and lentivirus (in vitro) both carry RNA vectors.	In vitro: over 60% In vivo: unreavled	Reduction in cccDNA and other HBV‐relevant duplication and translation factors	[69]
HBV concentrated 100‐fold from the culture medium of HepAD38 cells or HepG2.2.15 cells	HBx2 (2871–2893), HBx4 (2827–2849)	HepG2 cells expressing NTCP	Lentivirus‐carried sgRNA and CW‐Cas9 vectors (in vitro)	In vitro: over 80%	The DNA of HBV is 90% cleaved	[42]
HCV	miR‐122 locus (hcr)	Huh‐7 cells	AAV‐carried Cas9/sgRNA vector with the homologous recombination prototype pSSV9‐hcr‐donor‐shmiRHCV318 (in vitro)	In vitro: nearly 30%	Anti‐HCV shmiRNA expression following site‐specific integration, elimination of a full‐length reporter virus and a sub genomic HCV replication unit	[70]
HBV1.05	DNA polymerase κ (POLK): sgPOLK‐1 (5′‐CTTCTCCTTTGTGCTATCCA‐3′), sgPOLK‐2 (5′‐GATGATCTTCTGCTTAGGAT‐3′)	HepG2 cells expressing NTCP	sgRNA vector carried by lentivirus and CW‐Cas9 plasmid carried by Lipofectamine (in vitro)	Unpresented	Prevention of rcDNA reversion into cccDNA, a > 50% reduction of cccDNA synthesis	[71]
HBV1.3	Core, ORF S, X: sgB1‐sgB9, polymerase	Mouse models with hydrodynamic infusion of 1.3× HBV vectors, HepAD38 cells	Lipid‐like nanoparticle (LLN) carried Cas9 mRNA/sgRNA (in vitro and vivo)	Unshown	Reduction of all HBV viral load measures and stimulation of indels in the HBV DNA	[72]
HBV1.1	CP‐URR, S5, ORF S4, CP‐BCP, XP	HepG2.A64 (CCTCC C 201163) cells	Lipofectamine carried PX459 (in vitro)	Unshown	Complete elimination of HBV cccDNA and the full‐length integration of HBV DNA	[73]
HBV1.2 and HBV1.3	X, C1, ORF S, P	HepG2 cells expressing NTCP, Huh‐7 cells, HepAD38 cells, 1.2× HBV vectors infused hydrodynamically into mouse models	Lipofectamine carried PX458 (in vitro) and HDI (in vivo)	Unshown	Integrated action to halt HBV replication and eliminate the HBV genome	[74]
HBV1.3 and HBV1.2	Sa1, Sa2, Sa4	HepAD38 cells, Huh7 cells, HepG2.2.15 cells, 1.2× HBV vectors infused hydrodynamically into mouse models	Lipofectamine carried PX601 (in vitro), AAV and HDI (in vivo)	In vivo: unshown In vitro: 28.3%	Reduction in HBV DNA, HBsAg and pgRNA	[75]
HBV1.3	P1, XCp, HBV‐RT	HepG2 cells expressing NTCP, HepG2.2.15 cells	Delivery of PX330 by high‐capacity adenovirus (HCAdV) (in vitro)	In vitro: 37.4%	The administration of indels in the genome of HBV, Reduction in antigen formation of HBV and breakdown of cccDNA	[76]
HBV1.28	C: 21 gRNAs, ORF S, X, P	HBV‐Tg mice, HepG2 cells, HepG2.2.15 cells	rAAV type 8 carried PX601 (in vitro and in vivo)	In vivo: 41.05% In vitro: unshown	Decline in liver‐cell HBcAg, HBV DNA, serum HBsAg and HBeAg levels	[75]
HBV1.1 and HBV1.5	C: multiple gRNAs With various Cas9 types, ORF S, X, P	HepG2 cells	The transfection of NmCas9, SpCas9‐EGFP, StCas9 or FnCas9/gRNA vector via nucleofection (in vitro)	In vitro: over 85%	Over 90% HBV cccDNA degradation, About 60% prevention of HBV replication	[77]
HBV1.2	gHBV2, gHBV1	Huh7 cells, HepAD38 cells	PX458 carried via endogenous exosomes (in vitro)	Unshown	Blockage of HBV replication	[76]
HBV concentrated from the culture medium of HepAD38 cells	Human apolipoprotein E (apoE)	HepG2 cells expressing NTCP, HepAD38 cells	Lipofectamine‐carried Cas9/sgRNA vector (in vitro)	Unshown	Over 80% decrease of HBV synthesis and over 90% lessening of HBV infection	[78]
HBV1.3	ORF S, P	HepG2‐NTCP‐C4 cells, HepG2.2.15 cells, Huh‐7 cells	Lipofectamine‐carried sgRNA vectors pLenti‐FNLS‐P2A‐Pur (BE3) and pLenti‐BE4Gam‐P2A‐Pur (BE4) (in vitro)	In vitro: approximately or greater than 50%	Deactivation of integrated HBV DNA and cccDNA, Blockage of HBV gene expression	[79]
HBV concentrated from the culture medium of HepG2.2.15 cells	ORF S (crRNA)	HepG2‐NTCP‐30 cells	LNP carried Cas9/sgRNA RNP and ss‐ON complex in a microfluidic system (in vitro)	Unshown	Reduction in HBV cccDNA and DNA with 80% and 60%, respectively	[80]

## 6 CRISPR/Cas9 DNA‐Targeting Systems in HBV and HCV

DNA‐targeting nucleases such as Cas9 and derived base editors have been widely applied to HBV, a DNA virus, but are inherently unsuitable for HCV, which is a pure RNA virus with no DNA stage. For HBV, multiple studies have demonstrated that Cas9 can cleave HBV DNA, including covalently closed circular DNA (cccDNA) and integrated HBV DNA, thereby reducing viral markers [81]. For example, Stone et al. (2020) delivered adeno‐associated virus (AAV) vectors encoding *Staphylococcus aureus* Cas9 and HBV‐specific single‐guide RNAs (sgRNAs) into liver‐humanised mice with chronic HBV infection. They detected gene editing in the treated livers of 5 out of 8 mice and observed improved survival of human hepatocytes, along with a trend toward reduced total liver HBV DNA and cccDNA. These results suggest that Cas9‐mediated gene editing can reduce HBV persistence and enhance cell survival, although the reductions were modest and not all animals exhibited editing. In cell culture, Cas9 combined with dual or multiplexed guide RNAs has similarly suppressed HBV genome accumulation in chronically infected or transgenic cell lines by inhibiting cccDNA and antigen expression [82]. In contrast, because HCV does not produce a DNA intermediate, SpCas9 and SaCas9 cannot directly target the viral genome. One exception is the unusual *Francisella novicida* Cas9, which can be programmed to bind RNA; however, this approach remains largely experimental and is not widely used therapeutically [24].

Base editors, which are Cas9‐derived enzymes that introduce point mutations without causing double‐strand breaks, have also been tested on HBV. Smekalova et al. (2024) used a cytosine base editor (CBE) with two guide RNAs to introduce stop codons into HBV genes. They delivered base‐editing mRNA and guide RNAs via lipid nanoparticles (LNPs) in an HBV mini‐circle mouse model [83]. This single treatment resulted in more than a 3 log_10 reduction in serum HBV DNA and over a 2 log_10 decrease in HBsAg; 4 out of 5 mice lost detectable HBsAg [83]. In vitro, the same study demonstrated that base editing of HBV in HepG2‐NTCP cells and primary human hepatocytes strongly suppressed viral markers and prevented viral rebound after drug withdrawal. These findings demonstrate that Cas9‐based base editors can inactivate both covalently closed circular DNA (cccDNA) and integrated (HBV) DNA by introducing lethal point mutations [83]. However, such base‐editing approaches do not have a direct target in HCV, as HCV lacks a DNA intermediate. In summary, DNA‐targeting CRISPR systems (including Cas9 nucleases or base editors) apply to HBV, enabling disruption or mutation of cccDNA, but not to HCV. These systems have been used to cleave HBV cccDNA and integrated DNA in cell lines and animal models. For example, Stone et al. (2020) employed AAV‐delivered SaCas9 in humanised mice, which improved human hepatocyte survival and tended to reduce HBV DNA and cccDNA levels. Base editors convert cytosine to thymine (C → T) or adenine to guanine (A → G) without inducing DBSs [82]. Smekalova et al. (2024) used lipid nanoparticle (LNP) delivery of cytosine base editors (CBE) combined with guide RNAs in mice, achieving a sustained approximately 3‐log reduction in HBV DNA and loss of hepatitis B surface antigen (HBsAg). Similarly, base editing of HBV in cell cultures effectively silenced viral gene expression [83].

## 7 RNA‐Targeting CRISPR Systems (Cas13) in HBV and HCV

RNA‐guided RNases, such as CRISPR‐Cas13, can specifically target RNA viruses and viral transcripts. Cas13 enzymes (including Cas13a, Cas13b, Cas13d, etc.) have been employed to cleave (HCV) genomic RNA and (HBV) mRNAs. For HCV, Cas13a has been demonstrated to effectively knock down the viral genome [84]. Ashraf et al. (2021) designed Cas13a with guide RNAs targeting the conserved internal ribosomal entry site (IRES) of HCV [85]. In infected Huh‐7.5 hepatoma cells, Cas13a significantly reduced HCV RNA levels and reporter expression with negligible cytotoxicity. This study reported that Cas13a efficiently targets HCV in vitro by degrading HCV RNA, thereby potently suppressing HCV replication in cell culture and providing proof of concept for an RNA‐targeting antiviral strategy. For HBV, a DNA virus, Cas13 can target viral RNAs, including pregenomic RNA and mRNAs [85]. McCoullough et al. (2024) programmed PspCas13b to target multiple regions of HBV pregenomic RNA in HepG2‐derived models. They found that HBV‐targeting Cas13b guide RNAs strongly suppressed HBV replication and protein expression in mammalian cells by up to 96%. In both a stable HBV cell line and an infection model, HBV RNA and antigens were similarly reduced. In vivo, hydrodynamic injection of plasmids encoding Cas13b and guide RNAs into mice resulted in a 50% reduction in serum hepatitis B surface antigen (HBsAg) [85]. Furthermore, McCoullough et al. demonstrated (LNP)‐mediated delivery of Cas13b mRNA and guide RNAs into cells, achieving an 87% decrease in secreted HBsAg from a human hepatoma cell line. These results confirm that Cas13b can be harnessed to degrade HBV transcripts, thereby reducing viral DNA replication and antigen levels. Notably, Cas13 targeting of HBV transcripts may complement DNA‐targeting strategies by eliminating viral RNA and proteins. Summary: Targets single‐stranded RNA (ssRNA) [85]. Ashraf et al. (2021) used Cas13a with guide RNAs targeting the HCV IRES in Huh‐7.5 cells, significantly inhibiting HCV replication in vitro, suggesting Cas13a as a programmable antiviral against HCV RNA [85]. McCoullough et al. (2024) used Cas13b with guide RNAs against HBV pregenomic RNA and mRNAs, suppressing viral replication by up to approximately 96% in cells and reducing HBsAg by about 50% in vivo. LNP delivery of Cas13b similarly reduced HBsAg by 87% in a cell model. Reason: The text was revised to improve clarity, readability and technical accuracy. Redundant phrases were removed, and terminology was standardised. Both HBV and HCV can be targeted by RNA‐guided Cas13 effectors: HCV at the genome level and HBV at the level of its RNA transcripts [85] (A Cas9 ortholog, FnCas9, can also be engineered to bind RNA and has been reported to inhibit HCV in cells; however, the primary RNA‐targeting research for HCV has utilised Cas13) [24, 85].

## 8 CRISPR/Cas9 Delivery for HBV and HCV Infection Therapy

The challenges of intracellular transport continue to impede the therapeutic applications of the CRISPR/Cas9 system. Numerous proof‐of‐concept experiments using CRISPR/Cas9 to target HBV in mouse models have employed hydrodynamic tail‐vein injection, a technique with limited clinical utility [86] demonstrated that lentiviral vectors utilising CRISPR/Cas significantly decrease the replication of HBV in vitro. However, lentiviral delivery in vivo is ineffective in mature liver cells [87, 88]. Adenoviral plasmids guided by CRISPR/Cas9 can facilitate gene modification; however, their use has been limited by harmful immunostimulation [89, 90].

Various challenges arise with gene editors and the plasmids used to deliver CRISPR/Cas nucleotide sequences, including immune responses that may cause toxicity and reduce treatment efficacy [90, 91, 92]. Infectious hepatocellular conditions may require multiple injections of viral or non‐viral plasmid formulations before administering HBV‐targeting CRISPR/Cas genes. However, the therapeutic effectiveness of subsequent doses is expected to diminish due to the immune system's response to the genetic modifiers. Several researchers, including [92, 93, 94, 95], endorse the use of non‐viral carriers for the delivery of single‐guide RNAs (sgRNAs) and chemically modified messenger RNA (mRNA) encoding Cas9. Chemical modifications can enhance the stability of mRNA, making it less susceptible to detection and degradation by the innate immune system. Utilising RNA instead of DNA in therapeutic applications offers several advantages: the only required step is the translocation of mRNA to its target within the cytoplasm, and there is no risk of harmful effects resulting from recombination with the host genome.

The short half‐life of RNA enables gene editors to produce their products with greater precision and timing, thereby reducing the likelihood of non‐specific effects [96]. An innovative technique for gene editing in cultured cells and the inner ear of live mice was introduced through the administration of Cas9 protein and sgRNA complexes. Despite the promising results to date, extensive investigation is required to understand the implications of dose control and repeated administrations. Many gene therapy approaches have utilised transgenic adeno‐associated viral vectors, or rAAVs. These viral vectors are versatile, safe and exhibit low immunogenicity, posing minimal risk. The maximum transgene capacity of single‐stranded AAVs (ssAAVs) is approximately 4.6 kb. The vectors have limited data storage capacity due to the inclusion of the SpCas9 cassette and conventional sgRNA sequences. This limitation has been addressed by incorporating SpCas9 and sgRNA sequences into separate plasmids [90, 97] shown that the dual‐lobed architecture of SpCas9 can be efficiently utilised in the intein‐mediated protein splicing mechanism to recombine two AAVs into a seamless, full‐length SpCas9.

A primary focus of research has been the discovery of truncated Cas9 variants suitable for seamless integration into AAVs. This interest stems from the existence of numerous bacterial strains that possess CRISPR/Cas systems [98]. In 2015 [99], discovered that it may serve as an alternative to SpCas9. The 3159 bp coding sequence is approximately 1 kilobase shorter than the open reading frame of SpCas9. Similar to SpCas9, SaCas9 efficiently and selectively facilitates targeted insertions and deletions. Delivery via recombinant adeno‐associated viruses (rAAVs) resulted in a 40% increase in mutations of native hepatic genes. This ortholog is not limited to Lachnospiraceae; it has also been identified in *Streptococcus thermophilus* and *Neisseria meningitidis* [100, 101].

This study highlights the potential of adeno‐associated viruses (AAVs), including those utilising the CRISPR/Cas system, as a promising approach for treating HBV infection [102] AAVs may successfully induce transgenic expression lasting for more than a year. This could potentially provoke humoral immune responses and increase off‐target effects [90, 103]. The use of vectors and inducible promoters to modulate the synthesis of Cas9 and sgRNA cassettes is investigated. Guides in “self‐restricting” vectors specifically target Cas9 or adjacent sequences, thereby limiting the duration of gene editor expression. Utilising self‐target mismatches in Cas9 facilitates the inactivation of Cas9‐encoding DNA and triggers cccDNA cleavage. Reducing the active lifetime of the nuclease in this manner may decrease the likelihood of off‐target modifications. Numerous studies have focused on modifying existing wild‐type AAV sub‐genotypes to enhance transgene expression and hepatotropism in liver cells. Viral capsids were genetically modified using “shuffling” and in vivo evolution [104, 105]. These methods may enhance vector efficiency by reducing ubiquitin‐mediated degradation, as demonstrated by [106]. The co‐production of components, including non‐toxic phosphatases, may enhance the formation of subsequent ssAAV sequences, thereby preventing host proteins from modifying AAV DNA [107]. Substantial improvements have been achieved in the delivery of RNA encoding CRISPR/Cas components using non‐viral vectors. Many studies have aimed to identify methods to enhance formulations in terms of efficacy, safety, immunological response and the reduction of non‐specific interactions [93, 108, 109]. Research on both non‐viral and viral vectors is currently flourishing. Although it is an ambitious goal, delivering therapeutic sequences to every HBV‐infected cell in the liver is achievable.

## 9 Challenges in the Use of CRISPR/Cas9 Technology in Clinical Settings for the Treatment of HBV and HCV Infections

Before the therapeutic use of the CRISPR/Cas system, several significant challenges must be resolved. Accurate targeting of viral sequences requires the elimination of host cellular sequences [110, 111]. Specialised gene editors can effectively remove viral DNA sequences from chronic HBV infections, even though these sequences are often irreversibly integrated into the host genome [26, 112]. The inserted sequences and integration sites can vary significantly. The integration of HBV DNA may lead to genomic instability and harmful changes in host cells; however, our understanding of this process remains limited. Efficient delivery of potential therapies to affected cells is a major challenge that must be addressed [113].

## 10 Clinical Translation Issues With CRISPR/Cas9 Technology for Treating HBV and HCV Infections

The use of the CRISPR/Cas system in therapeutic applications depends on overcoming several significant challenges. Accurate identification of viral targets requires the elimination of host cellular sequences. Specialised gene editors can remove viral DNA sequences integrated into the host genome during persistent HBV infections [113]. The sites of integration and the incorporated sequences can differ significantly [114]. The breakage of integrated HBV DNA can cause genomic instability and harmful alterations in host cells; however, our understanding of these processes remains limited. Additionally, effectively delivering potential therapies to affected cells is a critical challenge that must be addressed [115].

## 11 The Specificity of CRISPR/Cas9 Targeting HBV and HCV Sequences

The effective application of the therapeutic CRISPR/Cas9 system depends on minimising unintended modifications in the normal regions of the host genome caused by off‐target effects. Detecting rare genetic alterations in small clusters of hepatic cells is challenging but essential to prevent potentially catastrophic outcomes such as hepatocarcinogenesis. In efforts to inactivate HBV, researchers utilised the previously characterised Cas9 from *Streptococcus pyogenes* (SpCas9). This Cas9 variant can tolerate mismatches in the single‐guide RNA (sgRNA) [116]. According to [117], a single sgRNA can bind to over 150 locations in the human genome, potentially causing mutations and chromosomal instability [68, 89, 118], found that different combinations of short genomic RNAs can inhibit the replication of herpes simplex virus (HSV). However, multiplexing sgRNAs increases the likelihood of off‐target effects, which is a significant drawback. By elucidating the mechanisms that regulate CRISPR/Cas interactions with their targets, several researchers have identified sgRNAs with a low probability of off‐target effects [43, 67, 69, 116, 119, 120] are among the few studies that have employed such approaches to inhibit the replication of HCV and HBV. Numerous HBV studies have evaluated non‐specific mutations using advanced next‐generation sequencing (NGS) or by examining non‐specific DNA breakage [121, 122, 123]. Prediction methodologies may exhibit a bias that underrepresents the extensive genomic variability of sgRNA, thereby confounding the assessment of off‐target mutations [123]. Progressing approaches are more innovative, sophisticated and unbiased [117, 123], Di‐Seq, GUIDE‐Seq, BLESS, integrase‐deficient lentiviral vectors (IDLV) and Di‐Seq each have distinct advantages.

The advancement of genome editing tools with therapeutic potential has been characterised by the development of increasingly precise CRISPR/Cas systems [124]. A refined and straightforward technique has been developed for synthesising truncated small RNAs (tru‐gRNAs) with nucleotide chains shorter than 20 nucleotides by removing unnecessary 5′ nucleotides. This approach is based on the premise that adding nucleotides to the 5′ terminus of the guide RNA may reduce destabilising mismatches in an RNA/DNA duplex. Subsequent studies have linked FokI single‐strand endonuclease domains to dCas9—catalytically inactive Cas9—to enhance specificity [101]. The increased specificity is attributed to the requirement of positioning two adjacent sgRNAs over an extended target region to facilitate duplex DNA cleavage. A dCas9‐FokI fusion was created to improve on‐target cleavage [117] found that these fusions not only co‐localise but also impair the target DNA through dimerization. Similarly, duplex cleavage was achieved by [99] using Cas9 nickases (nCas9s); these enzymes were initially limited to cleaving DNA on a single strand. However, the combination of two small guide RNAs (sgRNAs) proved crucial in overcoming this limitation [123] effectively used a nCas9 system to go against HBV; however [122], observed suppressed efficiency against HBV: (1) Finding appropriate target locations is more difficult than with conventional sgRNA/Cas9 systems; (2) sgRNA mismatches reduce antiviral effectiveness; and (3) the requirement for a trio of records in the targeted cells compromises delivery efficiency, especially when using carriers with limited capacity.

According to [125], In order to initiate target cleavage, SpCas9 must first engage with the (PAM) specifically the NGG sequence. Off‐target cleavage result occur due to sporadic occasional recognition of non‐canonical PAMs such as NGA [126]. The PAM interaction site of SpCas9 was modified to create variants that either demonstrate increased selectivity for NGG or recognise the extended PAM sequence NGCG [127]. PAM minimises global Cas9 binding to DNA on chromosomes over time, increasing its specificity. Analysis of Cas9 gene orthologs has further enhanced target specificity. The Cas9 gene derived from *Staphylococcus aureus* requires seed sequences and an expanded PAM (NNGRRT) due to its limited mismatch tolerance [99]. Utilising several orthologs of Cas9 with complex PAM sequences is an effective strategy to improve target specificity. *Streptococcus thermophilus* Cas9 recognises the PAM sequence NNAGAAW, while *Neisseria meningitidis* Cas9 recognises NNNNGMTT. Recent investigations by [52, 101], the architectures of DNA‐bound Cas9 have been clarified. This understanding has facilitated the systematic development of SpCas9 variants that bind to either the complementary or non‐complementary DNA strands with reduced energy expenditure. These modifications enable SpCas9 to accurately recognise and cleave its targets. Despite the therapeutic potential of Cas9 variants, their on‐target efficacy has been shown to be diminished [127]. Utilising methodologies that predict sgRNA effectiveness, evaluate off‐target effects and examine differences in RGN complexes would undoubtedly advance the process.

## 12 Conclusion

The CRISPR/Cas9 system, along with its newer adaptations, has become an invaluable tool for elucidating the mechanisms underlying viral infections, particularly those caused by hepatitis viruses. By directing CRISPR/Cas not only to the viral genome but also to host cell factors essential for viral replication, researchers have the potential to significantly reduce or even completely eradicate infections. These engineered CRISPR/Cas systems also play a crucial role in developing improved vaccine candidates and creating model cell lines for research purposes. By targeting cellular components that enhance viral replication, CRISPR technology opens new avenues for both prevention and treatment. As highlighted in this review, recent applications of CRISPR/Cas in combating HBV and HCV demonstrate innovative strategies that could lead to the next generation of therapeutic agents for both acute and chronic viral hepatitis.

## Author Contributions

**Meng‐Fan Li:** data curation, formal analysis. **Akmal Zubair:** conceptualization, writing – original draft, writing – review and editing, methodology, validation, formal analysis, project administration. **Safa Wdidi:** validation, project administration. **Shan He:** writing – review and editing, conceptualization.

## Funding

The authors have nothing to report.

## Ethics Statement

The authors have nothing to report.

## Conflicts of Interest

The authors declare no conflicts of interest.

## Data Availability

Data sharing not applicable to this article as no datasets were generated or analysed during the current study.
